# Yield Gaps in Wheat: Path to Enhancing Productivity

**DOI:** 10.3389/fpls.2019.01603

**Published:** 2019-12-06

**Authors:** Jerry L. Hatfield, Brian L. Beres

**Affiliations:** ^1^National Laboratory for Agriculture and the Environment, Agricultural Research Service, United States Department of Agriculture, Ames, IA, United States; ^2^Lethbridge Research and Development Centre, Lethbridge, Agriculture and Agri-Food Canada, Lethbridge, AB, Canada

**Keywords:** yield, wheat production, yield gap, weather, gap analyses

## Abstract

Wheat production is required to supply food for the world’s population, and increases in production will be necessary to feed the expanding population. Estimates show that production must increase by 1 billion metric tons to meet this demand. One method to meet future demand is to increase wheat yields by reducing the gap between actual and potential yields. Potential yields represent an optimum set of conditions, and a more realistic metric would be to compare actual yields with attainable yields, where these yields represent years in the record where there is no obvious limitation. This study was conducted to evaluate the yield trends, attainable yields, and yield gaps for the 10 largest wheat producing countries in the world and more localized yield statistics at the state or county level. These data were assembled from available government sources. Attainable yield was determined using an upper quantile analysis to define the upper frontier of yields over the period of record and yield gaps calculated as the difference between attainable yield and actual yield for each year and expressed as a percentage of the attainable yield. In all countries, attainable yield increase over time was larger than the yield trend indicating the technological advances in genetics and agronomic practices were increasing attainable yield. Yield gaps have not shown a decrease over time and reflect that weather during the growing season remains the primary limitation to production. Yield gap closure will require that local producers adopt practices that increase their climate resilience in wheat production systems.

## Introduction

Productivity of agricultural commodities throughout the world must increase in order to supply the food needs of the expanding population. Alexandros and Bruinsma (2012) estimated that by 2050, an additional 1 billion metric tons per year of cereals would be needed to meet the demand, which would require an increase in production from 2.1 to 3.0 billion metric tons. This requires that we either increase yield of crops through closing the yield gap between the potential and actual yields or by increasing the potential yield of crops. Evans and Fischer (1999) introduced the concept of potential yield in crops and the value of considering potential yield in evaluating progress of crop management programs. Most would argue that decreasing the yield gap is more achievable than increasing the potential yield. If we assume that the potential yield can be described as 

(1) $$Y_{p}=Cx\;St\;x\;\varepsilon_{i}\;x\;\varepsilon_{c}\;x\;\varepsilon_{p}$$

where Y_p_ is the potential yield, St is the incident solar radiation, C is the fraction of photosynthetically active radiation in total solar radiation, ε_i_ the interception efficiency, ε_c_ the conversion efficiency of solar radiation into photosynthetic products, and ε_p_ the efficiency of the conversion of stored carbon into harvestable products. Long et al. (2006) conducted an in-depth analysis on these terms and concluded that ε_i_ and ε_p_ are near the theoretical maximum for agronomic crops while there is improvement in ε_c_ possible and potentially increase Y_p_ by 50%. This relationship was first described by Monteith (1977) to evaluate the efficiency of light capture by crops and understanding how light is efficiently captured by agronomic crops will pay dividends in increasing crop productivity. We can describe the yield gap as

(2) $$Y_{g}=\;Y_{p}-Y_{a}w$$

where Y_g_ is the yield gap and Y_a_ the actual yield. There have been several studies on the yield gap for a variety of crops that has led to the development of a global yield gap atlas (www.yieldgap.org) based on the application of the use of Y_p_ and Y_a_ as described by van Ittersum et al. (2013). Guilpart et al. (2017) provide a methodology for estimating Y_g_ at the cropping system level. The approach has demonstrated the utility of being able to quantify yield gaps for different crops and climates.

In their analysis of yield gaps for a variety of crops, Fischer et al. (2014) provided an in-depth assessment of the trends in yields for different crops in their megaclimatic regions and proposed that Y_p_ would represent yields with no limitations of water or nutrients of the best-adapted varieties. Evans and Fischer (1999) proposed this definition to provide a standard for comparison among experiments. Fischer et al. (2014) defined farm yield (Y_af_) as the crop yield reported at the field, district, regional, or national average, and attainable yield (Y_at_) is the yield achieved under economically optimal practices with minimal limitations due to the weather during the growing season. Fischer et al. (2014) proposed yield gaps (Y_g_) should be expressed as a percentage of Y_at_ because this metric would have more impact in evaluating the limitations to production rather than Y_p_.

In their analysis of wheat (*Triticum aestivum* L.) yields and Y_g_, Fischer et al. (2014) found that increasing Y_p_ is an important factor in increasing Y_a_ and that increases in Y_a_ are a result of improved agronomic practices and would require implementation of multiple practices. They proposed that increases in Y_p_ are associated with increased grain number, harvest index, grain weight, and total dry matter. Yield gaps in wheat are closing slowly because of the adoption of agronomic practices that enhance Y_a_ (Fischer et al., 2014). Yield gaps have been the focus of several studies on a range of agronomic crops. Lobell et al. (2009) evaluated Y_g_ and found in irrigated wheat, rice (*Oryza sativa* L.), and maize (*Zea mays* L.) that yields were near 80% of the Y_p_, and that weather was the major constraint on productivity variation during the growing season. Nuemann et al. (2010) evaluated yield gaps of global grain production and suggested that closing the yield gap would require a detailed understanding of the specific limitations for each region. Mueller et al. (2012) found nutrient and water management were key to closing the yield gap because yield variability was affected by fertilizer use, irrigation, and climate. This conclusion was also supported by Sinclair and Rufty (2012) where they observed crop yield increases were more closely associated with nitrogen and water than plant genetics. Ray et al. (2015) observed that climate variation explained one-third of the global crop yield variability and in some areas of the world over 60% of the yield variability could be attributed to climate variation. Grassini et al. (2013) cautioned that historical yield trends needed to be evaluated to determine their trends and potential plateaus. They reported that wheat yields since 1960 in northern Europe, e.g., France, the Netherlands, and United Kingdom, had plateaued while increases were still evident in Australia, China, and India. In developing countries, George (2014) argued that crop yields have not increased in proportion to the advances in agronomic practices and there is much potential in productivity to be realized with adoption of improved practices.

Using yield gap analysis based on Y_at_ from country-level data in the Midwestern US, Hatfield et al. (2017) showed that Y_g_ on maize and soybean (*Gylcine max* L. Merr.) was related to July maximum temperatures, August minimum temperatures, and July–August total precipitation. Each of these climatic variables has a direct relationship to the physiological efficiency of these crops. However, in Great Plains wheat production, Hatfield and Dold (2018) found that Y_g_ was closely related to precipitation during the grain-filling stage and that temperatures were not a consistent limiting factor because wheat in these areas was not exposed to temperatures above the upper range for development. Analysis of the factors causing yield gaps can provide a valuable tool for assessing the limitations to productivity and improved management strategies to increase Y_af_. The objective of this study is to evaluate the yield gaps in the major wheat growing regions of the world using readily available data and to determine the limitations to productivity using yield gap analysis. The study serves as the foundation to develop strategies to decrease the yield gap for the major yield producing areas.

## Methodology

Data on wheat yields were collected from various sources to represent a range of scales. The primary data source was Food and Agriculture Organization Statistics (FAOSTAT) (www.fao.org/faostat accessed 3-June-2019) for the 10 highest-producing countries in the world. These countries are shown in **Table 1** with the area harvested and production for 2017. Data from 1961 through 2017 were extracted for the area harvested, yield, and total production. For the United States, state-level data were extracted for the top three wheat producing states, Kansas, North Dakota, and Washington, from the National Agricultural Statistics Service (nass.usda.gov, accessed June 3, 2019) for the period from 1950 through 2018. A more in-depth analysis was conducted for the top three producing counties in Kansas, Mitchell, Saline, and Sumner, with the data extracted from the NASS site for the period from 1950 through 2007. The area harvested and total production for these states and counties are shown in **Table 2**.

**Table 1:** Area of wheat production and annual production in 2017 and the average yield gap from 1960 to 2017 for the top 10 wheat producing countries.

Country	Area harvested in 2017 (ha)	Production in 2017 (metric tons)	Yield gap (1960–2017) and confidence limit
China	24,510,393	134,000,000	0.12 (0.01)
India	30,600,000	98,510,000	0.04 (0.005)
United States	15,210,680	47,370,880	0.12 (0.01
Russia Federation	27,517,354	85,863,132	0.21 (0.02)
France	5,464,689	36,924,938	0.15 (0.007)
Canada	9,035,993	31,818,744	0.24 (0.009)
Germany	3,202,600	24,481,600	0.00 (0.0009)
Australia	12,191,153	22,274,514	0.24 (0.01)
Pakistan	8,972,000	26,674,000	0.08 (0.007)
Turkey	7,662,273	21,500,000	0.12 (0.009)

Data extracted from FAOSTAT (www.fao.org/faostat).

**Table 2:** Area harvested and total production in 2018 for the three top producing states in the United States and the average yield gap from 1950 through 2018 and the area harvested and total production for the top three producing counties in Kansas and the average yield gap from 1950 through 2007.

State	County	Area harvested in 2018 (ha)	Production in 2018 (metric tons)	Yield gap (1950–2018)
Kansas		3,116,021		0.22 (0.03)
	Mitchell	82,069	187,653	0.36 (0.05)
	Saline	61,794	62,336	0.25 (0.04)
	Sumner	161,467	184,600	0.29 (0.05)
North Dakota		3,130,185	10,020,199	0.24 (0.03)
Washington		8,933,855	4,277,552	0.17 (0.02)

Data extracted from the National Agricultural Statistics Service (www.nass.usda.gov). Standard errors are shown in parentheses.

Analysis of the data was conducted across all yield records with the same approach. The process used quantile regression at the 95^th^ percentile (PROC QUANTREG in SAS, SAS for Windows v 9.3, SAS Institute, Cary, NC). This procedure was followed to find the attainable yield (Y_at_) for all of the observed yield data for a country, state, or county. Use of this approach to obtain boundary lines was described by Webb (1972) and Cade and Noon (2003). This method was used by Egli and Hatfield (2014a, 2014b) and uses a statistical method to select the years at the upper frontier of the record across the observed period of record to determine Y_at_ for different counties in the Midwest. These Y_at_ yields are assumed to represent the years in which weather was not a limitation to production. The yield gap is calculated as

(3) $$Y_{g}=\left(Y_{at}-Y_{a}\right)/Y_{at}$$

where Y_at_ is the attainable yield obtained from the quantile statistical analysis. Equation 3 provides the fraction yield gap. For each year, Y_at_ and Y_g_ are computed and an average of the Y_g_ computed across the total record. Data are presented to show the yield trend and Y_at_ and the temporal trend of Y_g_ for the 10 countries with the largest production since 1960. To obtain the yield trend line in the Y_a_ values, we used linear regression through the observed data with (PROC REG from SAS).

## Results and Discussion

### Yield Trends and Attainable Yield

In this analysis, we focused on two metrics from the yield record, the Y_at_ and Y_a_ values. The assumption made was that the slope of the Y_at_ line would represent the technology increase for a given country, while the slope of Y_a_ would represent the ability of the country-level yields to increase given the combination of technology and weather within the growing season.

Wheat yields in all countries have shown a continual increase since 1960. There is variation among the countries for both the slopes in the Y_at_ and Y_a_ values (**Table 3**). The differences between the two values for a given country reveal that technological advances have increased more than the yield trend line. If technology was the only factor contributing to the yield trend, then the expectation would be for the slopes of the lines to be similar; however, the slopes were significantly different (ρ < 0.01) using simple T-test. The standard error of the estimates was computed for each regression analysis and was quite small for all of the countries we evaluated. The Y_g_ values are computed on an individual year, and although there may be differences in the production area within a given country, this would be negated by the estimated Y_at_ and Y_a_ observations within a given year because these values are from the same land area. Yield gap analysis at the country level could be independent of the production region unless there was a major shift in the production region from good to poorer soils or from rain-fed to primarily irrigated areas. To evaluate this, we found no significant relationship between the area under production and Y_g_ for a given year across any of the countries. The assumption would be that trends in country-level yields would be reflective of the technology adoption within the country.

**Table 3:** Comparison of the slope of actual yield trends to attainable yield changes for the top wheat producing countries.

Country	Slope of the actual yield increase (kg ha^−1^ year^−1^)	Slope of attainable yield increase (kg ha^−1^ year^−1^)
China	88.3 (1.8)	99.8 (2.0)
India	44.8 (1.14)	57.6 (1.2)
United States	25.6 (0.5)	27.7 (0.6)
Russia Federation	43.9 (1.2)	48.7 (1.25)
France	83.4 (1.1)	125.9 (1.3)
Canada	31.7 (0.6	41.8 (0.9)
Germany	93.4 (2.1)	112.0 (2.1)
Australia	15.2 (0.3)	21.4 (0.4)
Pakistan	38.9 (0.7)	41.8 (1.1)
Turkey	27.3 (0.7)	28.9 (0.9)

Standard errors are shown in parentheses.

**Figure 1:**
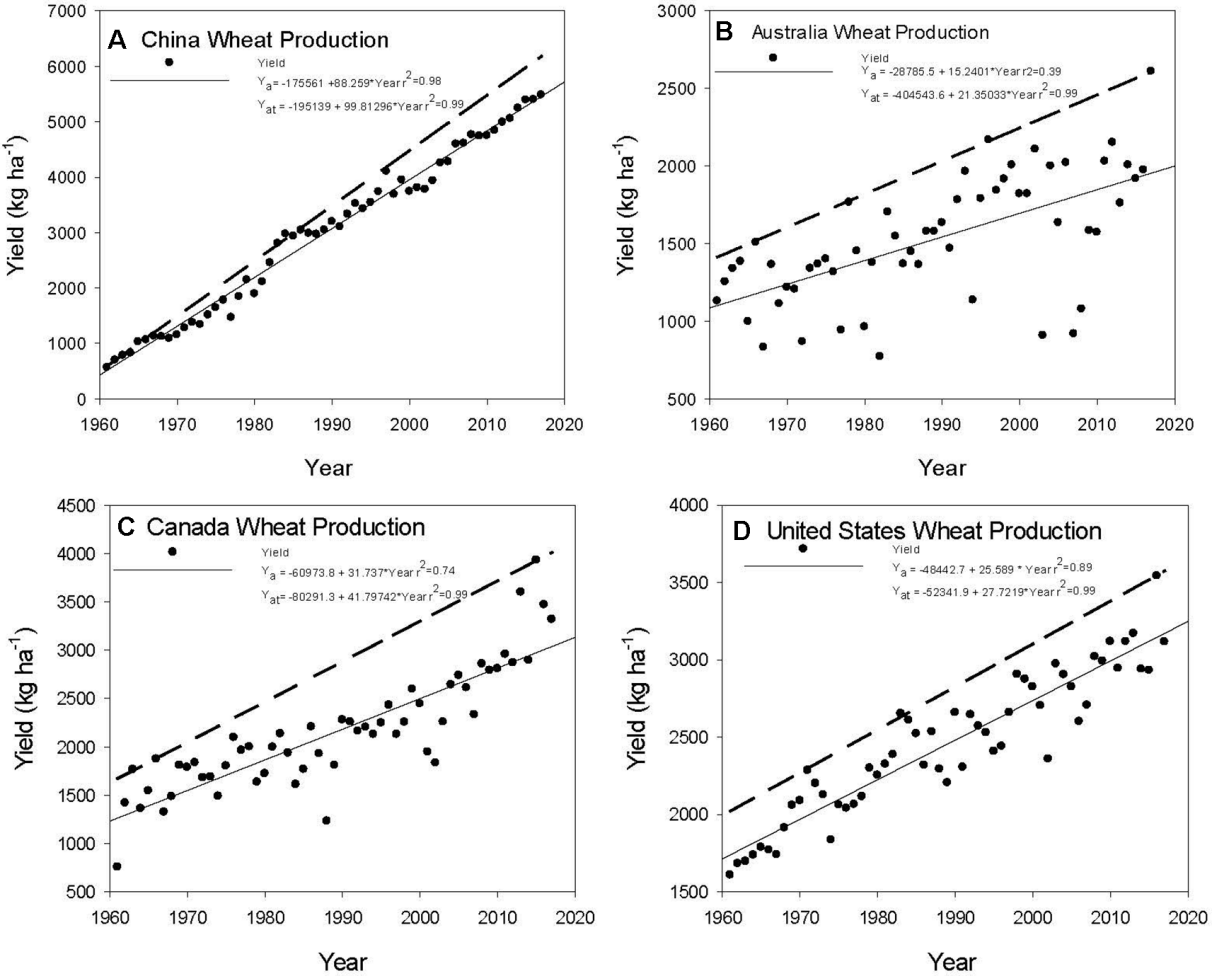
Yield trends and attainable yield for China **(A)**, Australia **(B)**, Canada **(C)**, and United States **(D)** from 1960 through 2017. Data from FAOSTAT (www.fao.org/faostat).

There are differences among countries. China wheat yields have shown an increase in yield; however, the slope of Y_at_ is 99.8 kg ha^−1^ year^−1^ while Y_a_ is at 88.3 kg ha^−1^ year^−1^. China has demonstrated the most consistent increase in wheat productivity since 1960, suggesting that wheat production has benefited from adoption of technology across the country (**Figure 1A**). If we compare the yield trends in Australia (**Figure 1B**), Canada (**Figure 1C**), and the United States (**Figure 1D**), these national scale yields show variability among the years with the Y_at_ trends exhibiting a larger increase than Y_a_. In the top wheat producing countries, the Y_at_ increase was greater than the Y_a_ trend (**Table 1**), with France and Germany showing the largest values in Y_a_ and Y_at_. These two countries have used a combination of advanced technology in managing the crop and have a climate that is ideally suited to wheat production because of the combinations of temperature and precipitation during the growing season. Technology adoption was suggested by Fischer et al. (2014) as being a significant factor in closing the yield gap.

### Yield Gaps

**Figure 2:**
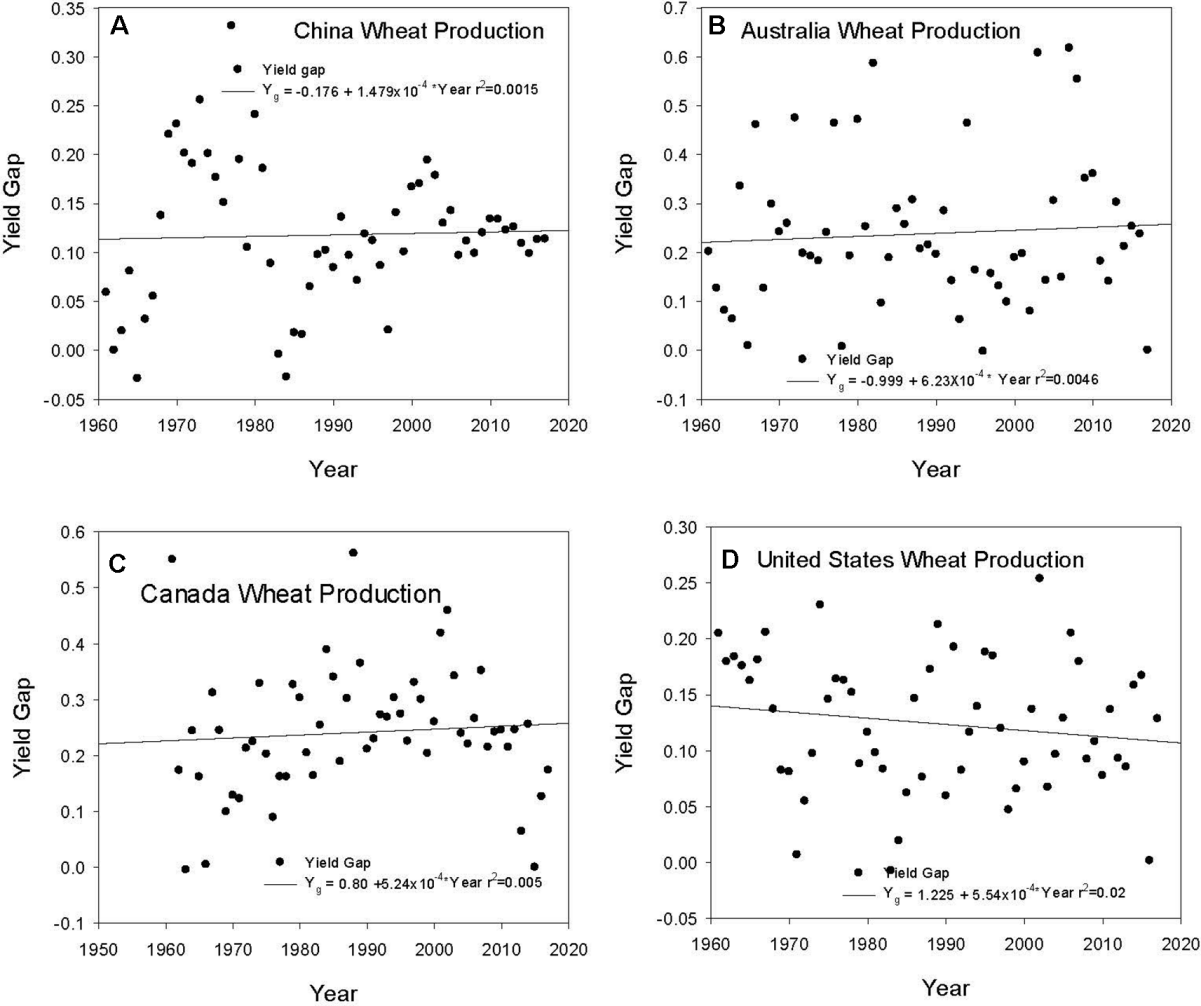
Yield gap trends from 1960 to 2017 for China **(A)**, Australia **(B)**, Canada **(C)**, and United States **(D)** using the data obtained from FAO (www.fao.org/faostat).

Each country has shown an increase in Y_a_; however, the Y_g_ has remained relatively constant over the years. The average Y_g_ for the period from 1961 through 2017 for the 10 top producing countries showed a range from 0.0 in Germany to 0.24 in Australia and Canada (**Table 3**). These are the average values calculated from the annual Y_g_ values. There is variation in Y_g_ across the years for all countries with no significant trend in closing the gap between actual and attainable wheat yields, e.g., China (**Figure 2A**), Australia (**Figure 2B**), Canada (**Figure 2C**), and the United States (**Figure 2D**). China is showing a decrease in the variation in Y_g_ in the last decade (**Figure 2A**); however, this has not impacted the overall Y_g_ trend. Australia exhibits the largest Y_g_ values, often exceeding 0.5, and there has been no change in the Y_g_ values since 1960 (**Figure 2B**). This can be attributed to the large variation in the meteorological conditions during the growing season related to the El Nino Southern Oscillation (ENSO) Index and years with large negative Southern Oscillation Index (SOI) values have the largest Y_g_ values and large positive values showed the smallest Y_g_ values. However, the scatter among all of the years showed the overall Y_g_ record was not significantly correlated with SOI values because other factors contributed to the inability of the wheat crop to achieve its potential. Canada has the same average Y_g_ value as Australia and has recorded Y_g_ values near 0.5; however, the past 4 years have shown very small Y_g_ values compared to the early record because of more favorable weather, e.g., slightly warmer temperatures and above-normal precipitation during the grain-filling period for the wheat producing regions. Yield gap values in the United States average 0.12 and show a trend toward decreasing Y_g_ values, but this trend is not significant. These observations of Y_g_ in these countries represent the range of yield gaps in wheat producing countries in the world. It is important to realize that national scale yields are comprised of many environments and soils and reflect the large-scale impacts of technology and the weather, and it is not possible to produce any assessment other than general trends.

### State- and County-Level Yields and Yield Gaps

**Figure 3:**
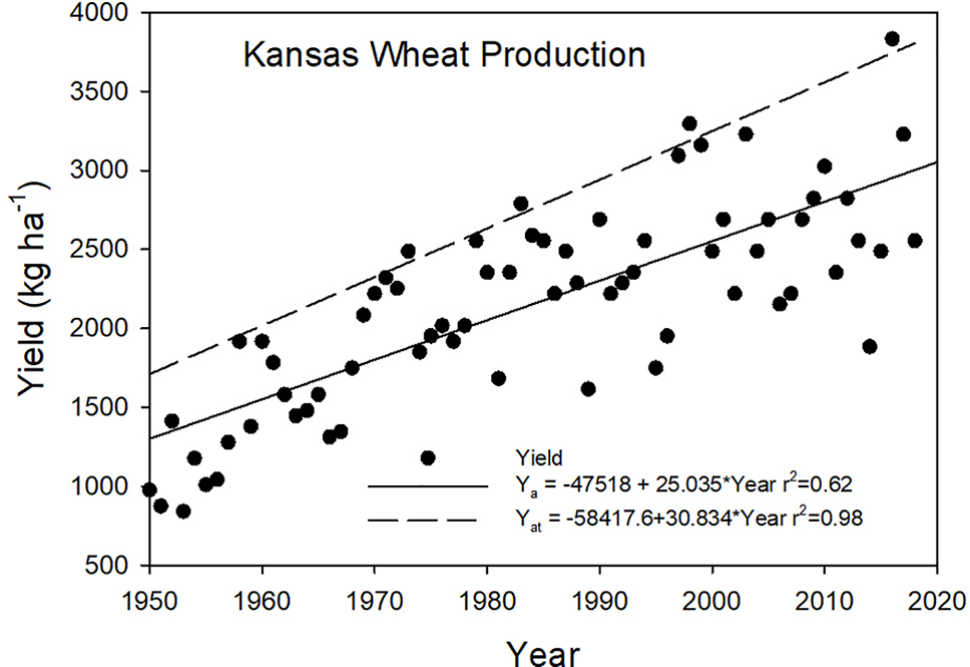
Winter wheat yield trends and attainable yields for Kansas from 1950 through 2018. (Data from www.nass.usda.gov).

To address the question of trends in more regional scale observations we extracted state-level data for the top three wheat producing states in the United States and computed their Y_at_ and Y_g_ values. The yield trends for Kansas show an increase in Y_a_ over time with no decrease in the Y_g_ values (**Figure 3**). We found the same results for North Dakota and Washington with increasing yields but no decrease in the Y_g_ values over time. To further refine the scale to the county level, the top three wheat producing counties in Kansas were selected, and they also showed the same pattern as the Kansas aggregate data. There were differences among the three counties in their average Y_g_ values (**Table 2**), with Mitchell county showing the largest Y_g_ average. The same results were observed by Hatfield and Dold (2018) when they examined Kansas, Oklahoma, and North Dakota wheat production and found Y_g_ values were related to the precipitation amounts during the grain-filling period. For these three counties, one weather event in 2007, a late spring freeze during heading was responsible for large Y_g_ values pf 0.64 (Mitchell), 0.76 (Saline), and 0.71 (Sumner).

**Figure 4:**
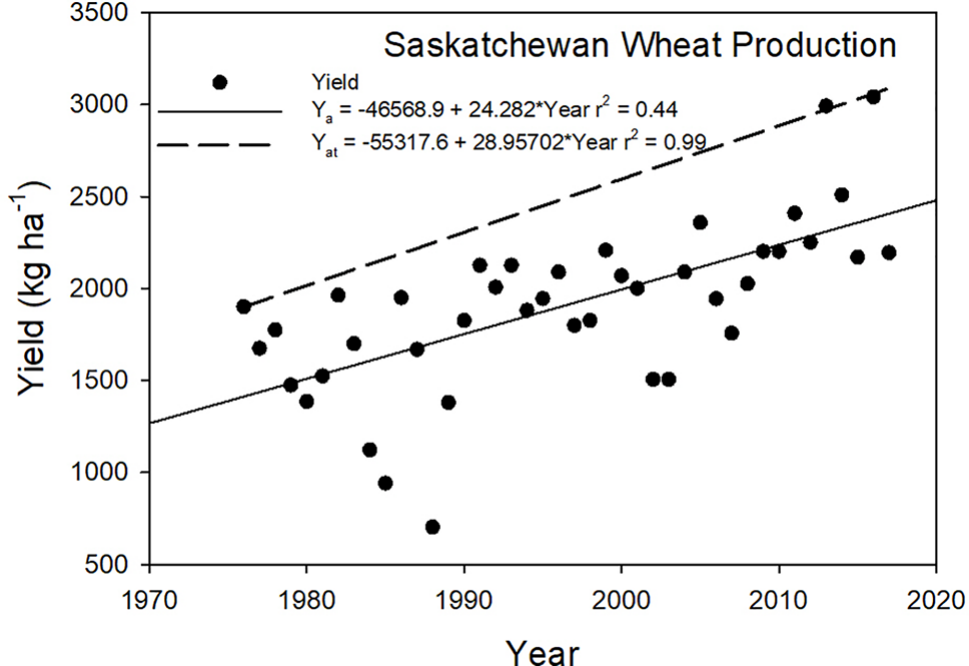
Spring wheat yield trends and attainable yield for Saskatchewan, Canada, from 1976 through 2017.

Saskatchewan is the top wheat producing province in Canada and shows the same trends as the whole country of Canada (**Figure 4**). There is a difference in Canadian wheat production with the growth of spring wheat varieties in the western provinces. The attainable yields exhibit a larger slope than the observed yield trends revealing that weather limitations on yields reduce the effect of technology. As we change the scale of the observed yield trends, there will be greater differences in the variation around the trend line because of the more local effects of weather and soil variations and their interactions. For example, within county-level yields, the impact of a drought or freeze could be quite large; however, at the statewide or country level, these effects may not be seen because the effects would not be evident across the large area.

### Yield Gap Trends

There is no discernable trend in the Y_g_ values across any scale we examined in this study. The Y_at_ values exhibit a larger increase than the Y_a_ trends, indicating that technology (agronomic and genetic) has increased the attainable yield and the potential yield; however, we are not closing the yield gap. The fact that the Y_g_ values have not decreased would suggest that weather remains the dominant factor limiting production around the world because the adoption of technology has provided for substantial yield increases across all countries. Since weather is the dominant effect on Y_g_, the challenge will be to determine how climate resilient a cropping system can be for a given region. This will require changes in the management practices as proposed by Hatfield and Walthall (2015) where they discussed the role of the genetics × environment × management (GxExM) concept in providing a framework for increasing productivity. The scale of yield data assembled from national, province, state, or county level provides an indication of the potential progress toward reducing the Y_g_ at a large scale. However, this scale doesn’t provide potential options for a producer to increase their productivity and reduce the yield gap. Evaluation of specific factors and potential management options for producers will have to be evaluated at a scale that represents the actual growing conditions. The analyses in this study were focused on the country-scale assessments to determine our progress toward decreasing the yield gap.

## Conclusions

The concept of yield gaps provides a framework for assessing the trends in yield for all crops. Across the top wheat producing countries of the world, there are differences in the progress for increasing yield. In France and Germany, the yield increase is near 100 kg ha^−1^ year^−1^, while in Australia, it is 15 kg ha^−1^ year^−1^, which can be attributed to a large difference in the variation in the climate between these two regions. There is also a major difference in the magnitude of the Y_g_ values between these two areas. Yields are more stable in the northern Europe environments compared to the Australian continent, also reflective of the weather variation among growing seasons. Evaluating smaller-scale yields, e.g., county, reveals that weather within the growing season is the dominant factor affecting yield gaps (Lobell et al, 2009). Technological advances have increased the attainable yields at a greater level than the yield trends, indicating that to close the yield gap, wheat producers will have to adopt practices at the local scale that will allow the technology improvements to be realized. These are local decisions made by individual producers; however, efforts to demonstrate how soil and agronomic practices that increase productivity could reduce the yield variation among years will pay dividends in closing the yield gap in wheat.

## Data Availability Statement

The datasets generated for this study are available on request to the corresponding author.

## Author Contributions

JH prepared the data analysis and JH and BB jointly prepared the paper.

## Funding

This research is supported from project 5030-11610-005-00D.

## Conflict of Interest

The authors declare that the research was conducted in the absence of any commercial or financial relationships that could be construed as a potential conflict of interest.

## References

[B1] Alexandros N. Bruinsma J. (2012). World agriculture towards 2030/2050. 2012 revision. ESA Working Paper No.12.03 FAO Rome http://www.fao.org/3/a-ap106e.pdf

[B2] Cade B. S. Noon B. R. (2003). A gentle introduction to quantile regression for ecologists. Front. Ecol. Environ. 1, 412–420. 10.1890/1540-9295(2003)001[0412:AGITQR]2.0.CO;2

[B3] Egli D. B. Hatfield J. L. (2014a). Yield gaps and yield relationships in central U.S. Soybean Prod. Syst. Agron. J. 106, 560–566. 10.2134/agronj2013.0364

[B4] Egli D. B. Hatfield J. L. (2014b). Yield and yield gaps in Central U.S. Corn Prod. Syst. Agron. J. 106, 2248–2256. 10.2134/agronj14.0348

[B5] Evans L. T. Fischer R. A. (1999). Yield potential: its definition, measurement and significance. Crop Sci. 34, 1544–1551. 10.2135/cropsci1999.3961544x

[B6] Fischer R. A. Byerlee D. Edmeades G. (2014). Crop yields and global food security: Will yield increase continue to feed the world? ACIAR Monograph 158 (Australian Centre for International Agricultural Research: Canberra). xxii+634 pp.

[B7] George T. (2014). Why crop yields in developing countries have not kept pace with advances in agronomy. Global Food Sec. 3, 49–58. 10.1016/j.gfs.2013.10.002

[B8] Grassini P. Eskridge K. M. Cassman K. G. (2013). Distinguishing between yield advances and yield plateaus in historical crop production trends. Nat. Commun. 4, 2918. 10.1038/ncomm3918 24346131 PMC3905725

[B9] Guilpart N. Grassini P. Sadras V. O. Timsina J. Cassman K. G. (2017). Estimating yield gaps at the cropping system level. Field Crops Res. 206, 21–32. 10.1016/j.fcr.2017.02.008 28515571 PMC5421155

[B10] Hatfield J. L. Dold C. (2018). Agroclimatology and wheat production: coping with climate change. Front. Plant Sci. 10, 103. 10.3389/fpls.2018.00224 PMC5826184 29515617

[B11] Hatfield J. L. Walthall C. L. (2015). Meeting global food needs: realizing the potential via genetics x environment x management interactions. Agron. J. 107, 1251–1226. 10.2134/agronj15.0096

[B12] Hatfield J. L. Wright-Morton L. Hall B. (2017). Vulnerability of grain crops and croplands in the Midwest to climatic variability and adaptation strategies. Climatic Change 146, 263–275. 10.1007/s10584-017-1997-x

[B13] Lobell D. B. Cassman K. G. Field C. G. (2009). Crop yield gaps: Their importance, magnitudes, and causes. Annu. Rev. Environ. Resour. 34, 179–204. 10.1146/annurev.environ.041008.093740

[B14] Long S. P. Zhu X. Naidu S. L. Ort D. R. (2006). Can improvement in photosynthesis increase crop yields. Plant Cell Environ. 29, 315–330. 10.1111/j.1365-4030.2005.01493.x 17080588

[B15] Monteith J. L. (1977). Climate and efficiency of crop production in Britain. Philos. Trans. R. Soc. Lond. B 281, 277–294. 10.1098/rstb.19770140

[B16] Mueller N. D. Gerber J. S. Johnston M. Ray D. K. Ramankutty N. Foley J. A. (2012). Closing yield gaps through nutrient and water management. Nature. 490, 254–257. 10.1038/nature11420 22932270

[B17] Neumann K. Verburg P. H. Stehfest E. Müller C. (2010). The yield gap of global grain production: A spatial analysis. Agric. Syst. 103 (5), 316–326. 10.1016/j.agsy.2010.02.004

[B18] Ray D. K. Gerber J. S. MacDonald G. K. West P. C. (2015). Climate variation explains a third of global crop yield variability. Nat. Commun. 6, 5989. 10.1038/ncomms6989 25609225 PMC4354156

[B19] Sinclair T. R. Rufty T. W. (2012). Nitrogen and water resources commonly limit crop yield increases, not necessarily plant genetics. Global Food Secur. 1, 94–98. 10.1016/j.gfs.2012.07.001

[B20] Van Ittersum M. K. Cassman K. G. Grassini P. Wolf J. Tittonell P. Hochman Z. (2013). Yield gap analysis with local to global relevance—a review. Field Crops Res. 143, 4–17. 10.1016/j.fcr.2012.09.009

[B21] Webb R. A. (1972). The use of boundary lines in the analysis of biological data. J. Hortic. Sci. 47, 309–319. 10.1080/00221589.1972.11514472

